# Using Next-Generation Sequencing and Bioinformatic Methods to Predict New Genes That May Be Regulated by CD47 in Oral Squamous Cell Carcinoma

**DOI:** 10.3390/cimb44050152

**Published:** 2022-05-17

**Authors:** Chung-Chih Tseng, Chen-Han Tsou, Shi-Ying Huang, Chia-Wei Wu, Tsung-Hua Hsieh

**Affiliations:** 1 Institute of Medical Science and Technology, National Sun Yat-sen University, Kaohsiung 80424, Taiwan; caviton@gmail.com; 2 Department of Dentistry, Zuoying Branch of Kaohsiung Armed Forces General Hospital, Kaohsiung 81342, Taiwan; chanhan@yahoo.com.tw; 3 College of Ocean Food and Biological Engineering, Jimei University, Xiamen 361021, China; johnhuang@jmu.edu.cn; 4 Department of Medical Research, E-Da Hospital/E-Da Cancer Hospital, I-Shou University, Kaohsiung 82445, Taiwan; snoopy79101@gmail.com

**Keywords:** CD47, next-generation sequencing, bioinformatics, OSCC

## Abstract

Oral squamous cell carcinoma (OSCC) is one of the most common cancers in the world, and the incidence and death rate of OSCC in men is twice that of women. CD47 is a ubiquitous cell surface transmembrane protein, also known as integrin-related protein (IAP). Previous studies have pointed out that CD47 can inhibit the growth of OSCC, but the detailed mechanism is not clear. This study aimed to explore the effect of CD47 gene expression profiles in OSCC. The OSCC cell lines, OECM-1 and OC-2, overexpressed CD47, and the expression profiles of mRNAs were analyzed through next-generation sequencing (NGS) with a bioinformatic approach. A total of 14 differentially expressed genes (DEGs) were listed. In addition, ingenuity pathway analysis (IPA) was used to analyze the molecular function (MF), biological process (BP), and cellular component (CC) network signaling. The human protein atlas (HPA) database was used to analyze gene expression and the survivability of human cancer. The results found that HSPA5, HYOU1, and PDIA4 were involved in the IPA network and when highly expressed, mediated the survivability of cancer. In addition, HSPA5 was positively and significantly correlated with CD47 expression (*p* < 0.0001) and induced by CD47-overexpression in the OECM-1 and OC-2 OSCC cancer cell lines. These findings provide important insights into possible new diagnostic strategies, including unfolded protein for OSCC-targeting CD47.

## 1. Introduction

Oral cancer is one of the most common cancers in the world. It is estimated that 657,000 new cases of oral and throat cancer are generated every year, and more than 330,000 people die. The incidence and death rate of oral cancer in men is twice that of women [1]. Normally, oral cancer is a group of head and neck cancers, formed on the mucosal surface of the lips, hard palate, posterior molar triangle, and mandible [2]. The most common oral cancer tissue type is squamous differentiated carcinoma, caused by epithelial mucosa, also known as oral squamous cell carcinoma (OSCC), which accounts for more than 90% of all oral cancers [3]. Oral cancer can be cured in its early stage by surgery, radiotherapy, and chemoradiation. However, most patients with OSCC already have advanced oral cancer when diagnosed. At this time, the treatment effect and prognosis are poor, and the mortality rate is high [4,5,6]. Studies have shown that the occurrence of oral cancer is related to multiple risk factors including tobacco use, drinking alcohol, betel chewing, human papillomavirus (HPV) [7], a weakened immune system, a lack of nutrition in the diet, and a poor lifestyle. In particular, betel chewing, excessive alcohol consumption, and smoking cause an increased incidence and mortality of OSCC [8,9].

CD47 is a ubiquitous cell surface transmembrane protein, also known as integrin-related protein (IAP), which belongs to the immunoglobulin superfamily (IgSF). It can interact with a variety of cell surface receptors including integrins (such as αvβ3, αIIbβ3, and α2β1), thrombospondin (TSP-1), and signal regulatory protein α (SIRPα), for the signal initiation related pathway [10,11]. For example, CD47 is the ligand of SIRPα, which is a transmembrane receptor of the immunoglobulin (Ig) superfamily with extracellular immunoglobulin-like domains and structural antigen receptors. In bladder cancer, CD47 initiates an immune checkpoint through the SIRPα signaling pathway [12]. In addition, CD47 can also be used as a receptor of TSP-1, which is secreted by several non-hematopoietic cells such as platelets, monocytes, and macrophages. CD47 is involved in a variety of physiological functions including leukocyte adhesion and migration, T cell activation, apoptosis, and phagocytosis [13,14,15]. Related studies have shown that the expression of CD47 increases in migrating hematopoietic stem cells (HSCs), which may provide protection from an important feature of macrophage killing [16]. Therefore, the expression level of CD47 can predict the possibility of HSCs being swallowed during circulation. More evidence indicates that CD47 is a common mechanism found in human solid tumor cells [17,18,19]. Through these mechanisms, tumor cells can protect themselves from phagocytosis, which results in proliferation and metastasis [20].

During immunotherapy, we found that chimeric antigen receptor (CAR)-T cells that bind CD47 antigen specifically kill different types of cancer including ovarian and pancreatic cancer cells [21]. Interestingly, a previous study also found that the effect of radiotherapy can be activated by targeted immunotherapy, as silencing of CD47 and HER2 eliminates radioresistant breast cancer cells [22]. In addition, previous studies have found that high expression of CD47 was detected in OSCC cell lines and in vivo results have shown that CD47 was significantly higher in OSCC than in proximal normal tissues. Furthermore, the poor prognosis in patients with OSCC has been connected to the CD47-SIRPα signaling pathway [23].

In the present study, we examined gene expression profiles by NGS and associated these data with gene network signaling through bioinformatic analyses of OSCC cell lines. We found that unfolded proteins HSPA5, HYOU1, and PDIA4 are induced by CD47 within the OSCC IPA network.

## 2. Materials and Methods

### 2.1. Cell Culture

Two human OSCC cell lines (OECM-1 and OC-2) were purchased from the American Type Culture Collection (ATCC, Manassas, VA, USA). The cancer cell lines were cultured in DMEM/F12 (Gibco, Courtaboeuf, France) supplemented with 10% fetal bovine serum (FBS) (Gibco) and 1% penicillin/streptomycin at 5% CO_2_ and in a 37 °C humidified incubator.

### 2.2. Plasmid Transfection

For plasmid transfection, the OSCC cell lines were seeded at a density of 1 × 10^5^ cell/well in a 6-well plate. After 24 h, 1 μg of the pcDNA3.1 (empty control), pcDNA3.1-CD47 (Addgene, Watertown, MA, USA, #65473) and HSPA5 plasmid (Addgene, Watertown, MA, USA, #27165) were incubated with TurboFect (Fisher Scientific, Waltham, MA, USA) transfection reagent according to the manufacturer’s instructions. TurboFect transfection reagent and plasmid were mixed and added to the cell.

### 2.3. RNA Extraction and qPCR

The total RNA was extracted from cell lines by the TRIzol reagent (Invitrogen, Carlsbad, CA, USA), and cDNA was synthesized using a cDNA Synthesis kit (Promega, Madison, WI, USA) according to the manufacturer’s instructions. The cDNA was PCR-amplified with the primers for CD47 (forward: 5′-AGA TCC GGT GGT ATG GAT GAG A-3′; reverse: 5′-GTC ACA ATT AAA CCA AGG CCA GTA G-3′), HSPA5 (forward: 5′-CTG GCA AGA TGA AGC TCT CC-3′; reverse: 5′-AAA ACC CGA CAG AGG GAC AT-3′), HYOU1 (forward: 5′- GAC TTC GGC ATC TGA GTG GT-3′; reverse: 5′-GCT CCC AAG TCC ACC ATT AC-3′), PDIA4 (forward: 5′- GCT CAG CTC CAG GGA GAG -3′; reverse: 5′- GAT GAT CTC CAC CCA CCT GT-3′). β-actin (forward: 5′-ATG ATA TCG CCG CGC TCG TCG TC-3′; reverse: 5′-CGC TCG GCC GTG GTG GTG AA-3′) was used as the reference gene for normalization. qPCR values were analyzed by the ABI-7500 system (Applied Biosystems, Foster City, CA, USA) and the values were calculated using the 2−^ΔΔCT^ method.

### 2.4. RNA-Seq Quantification

Differential gene expression (DEG) was analyzed by RNA-seq quantification following the protocol of Illumina (Genomics, Taipei, Taiwan). Briefly, Poly-T oligo-attached magnetic beads were used to purify the mRNA and synthesize double-strand cDNA, which was then sequenced on the Illumina NextSeq 500 platform, which obtained approximately 10 million reads for each sample. The gene expression profiles were analyzed and compared between the CD47-overexpressing and control OECM-1 and OC-2 oral cancer cell lines. All genes showing differential expression were further categorized using Gene Ontology (GO) and DEG report analysis.

### 2.5. Ingenuity Pathway Analysis

The IPA software (Ingenuity Systems, Redwood City, CA, USA) comprehensively explores DEGs to determine their biological function and signaling pathway. The DEGs were mapped to the IPA genetic network, then the score was calculated and sorted. The score was calculated by computing a statistic for each biological function according to a model that assigns random adjustment directions. The IPA software was used to conduct network analysis of CD47 downstream genes and to explore the underlying genetic networks.

### 2.6. Database for Annotation, Visualization and Integrated Discovery Analysis

DAVID analysis is a powerful tool for classifying genes through their function. It integrates data and calculates similarities using the global annotation map derived from multiple functional annotation databases including the Kyoto Encyclopedia of Genes and Genomes (KEGG) pathways and Gene Ontology (GO) biological processes.

### 2.7. Cell Viability Assay

Cell viability was analyzed using Cell Counting Kit 8 (CCK-8) (Dojindo, Kumamoto, Japan). The 1 × 10^4^ cells were seeded into 96-well plates, 24 h after transfection. Cells were incubated with 10 μL of CCK-8 solution at 37 °C for 1 h. The OD value was measured at 450 nm with a microplate reader (Multiskan GO, Thermo Scientific, Carlsbad, CA, USA).

### 2.8. Statistical Analysis

The gene expression was compared between cells with CD47-overexpression and normal cells. All statistical analyses were performed using the GraphPad Prism statistical software (GraphPad Software, San Diego, CA, USA).

## 3. Results

### 3.1. Gene Expression Profiling and Ovexpression of CD47 in OSCC Cell Lies

**Figure 1:**
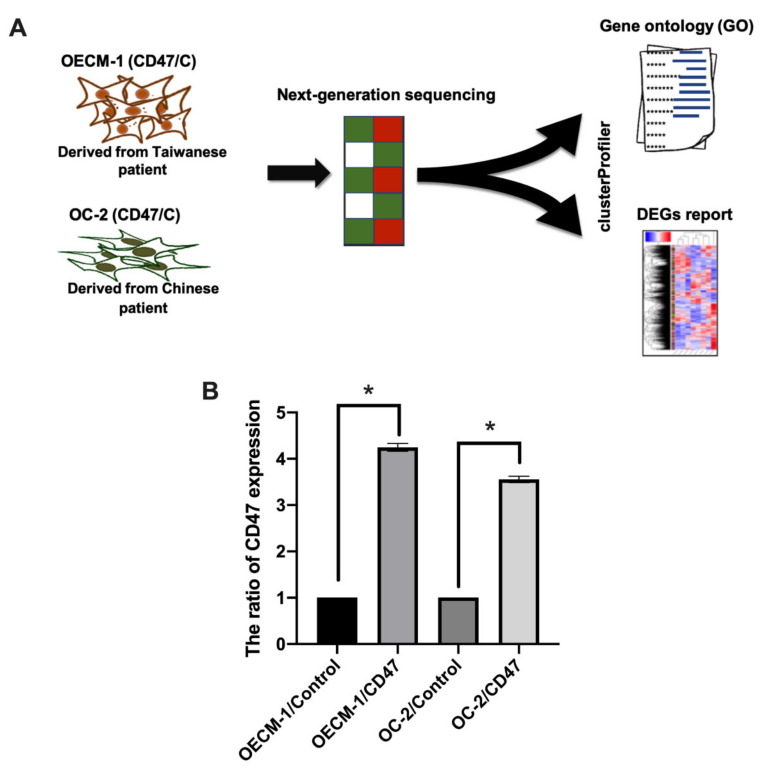
Investigation of CD47 expression in OECM-1 and OC-2 cells. (**A**) The flow chart shows the method of studying various gene expression changes with overexpressed CD47 in OECM-1 and OC-2 cells, through a gene expression array. The clusterProfiler tool was used to analyze bioinformatic approaches including gene ontology (GO) and DEGs. (**B**) Overexpression of CD47 was performed by transfecting cells with CD47 plasmid (1 μg), and CD47 expression was detected with qPCR and compared to the empty control. The data are presented as the mean ± standard deviation (SD) of three experiments. The data are presented as the mean ± SD of three experiments. * *p* < 0.05 vs. empty control; two-tailed Student’s *t*-test.

Previous studies showed that CD47-overexpression may regulate the growth process of oral cancer [23]. In this study, we mainly focused on Asian patients suffering from OSCC. OECM-1 cells were derived from the surgical resection of a primary tumor from a Taiwanese patient with a history of betel quid chewing. OC-2 cells were derived from a primary tumor of the buccal mucosa of a Chinese patient with a habit of alcohol drinking, betel quid chewing, and cigarette smoking [24,25]. We used Illumina NextSeq 500 sequencing and bioinformatic studies to analyze the genes in the OECM-1 and OC-2 cells and whether they were regulated by CD47 (Figure 1A). In addition, CD47 was overexpressed in OECM-1 and OC-2 cells (Figure 1B). The RNA was extracted from the cells and the gene profile was analyzed by NGS.

### 3.2. Gene Expression Profile in CD47-Overexpressing Oral Cancer

**Figure 2:**
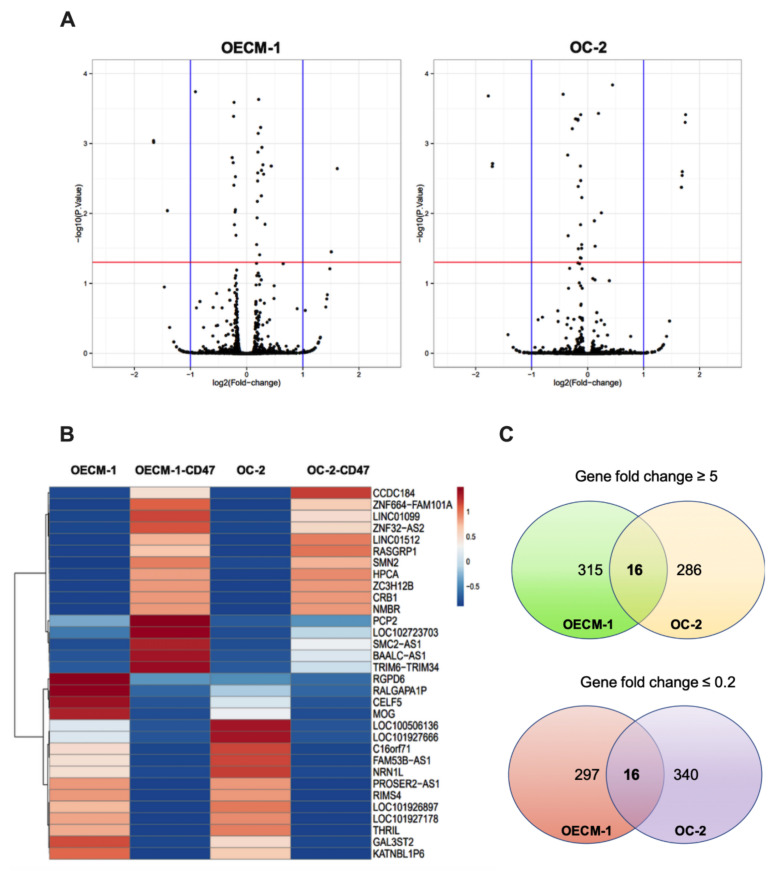
Fold changes in mRNA expression in CD47-overexpressing oral cancer cells. (**A**) A volcano plot showing the differentially upregulated (right panel) and downregulated (left panel) genes in CD47-overexpressing OECM-1 and OC-2 cell lines. (**B**) Heatmap visualization of the RNA sequencing analysis results showing differentially expressed genes with-log10 (*p* value) and ≥5-fold and ≤ 0.2-fold changes. (**C**) Venn diagram of genes shows that 617 genes were upregulated and 653 genes were downregulated due to CD47-overexpression in oral cancer.

Figure 2A shows the differentially upregulated (right panel) and downregulated (left panel) gene expression in CD47-overexpressing OECM-1 and OC-2 oral cancer cell lines. Genes with-log10 (*p* value) and ≥5-fold and ≤0.2-fold changes were chosen for further analyses (Figure 2B). Our results identified that the expression of 16 upregulated and 16 downregulated genes was found to be associated with CD47-overexpression in OECM-1 and OC-2 cells compared to the empty control (Figure 2C) (Table 1).

### 3.3. Biological Process Analysis of Differentially Expressed Genes in CD47-Overexpressing Oral Squamous Cell Carcinoma

**Figure 3:**
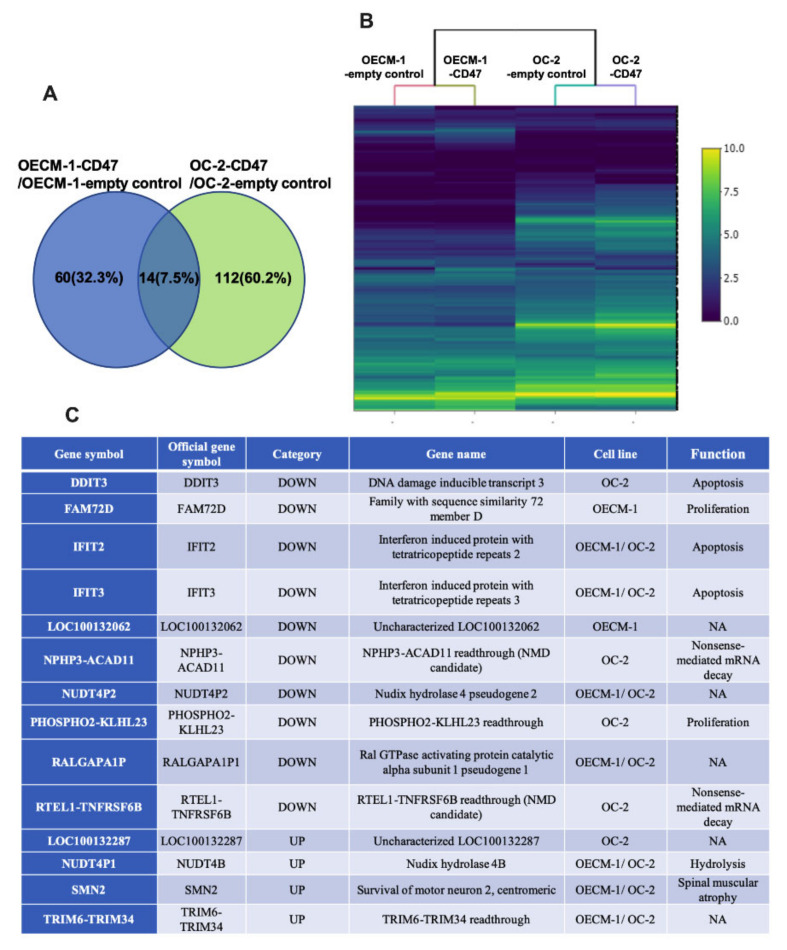
The function of differentially expressed genes in CD47-overexpressing oral cancer cells. (**A**) The number of DEGs in each comparison was determined by RSEM and EBSeq (PPEE ≤ 0.05). Fourteen genes were found to be involved in molecular functions, biological processes, and cellular components. (**B**) Heatmap demonstrating the expression profiles of union sets. The color bar on the right side indicates the log2 fold changes. (**C**) The list presents the 14 associated genes, including the DEGs in OECM-1 and OC-2 cells and their functions in biological processes. NMD: nonsense-mediated mRNA decay; Readthrough: continuous transcription; NA: not applicable.

Simultaneously, we executed an intergroup comparison analysis of DEGs in each comparison, determined by RNA-seq by expectation-maximization (RSEM) and differential expression analysis at both gene and isoform level using RNA-seq data (EBSeq) (posterior probability of equal expression (PPEE) ≤ 0.05). In total, 14 genes were commonly identified as DEGs in OECM-1 and OC-2 oral cancer cells with CD47-overexpression (Figure 3A). Furthermore, we used a heatmap to display the expression profiles of union sets (Figure 3B,C).

**Figure 4:**
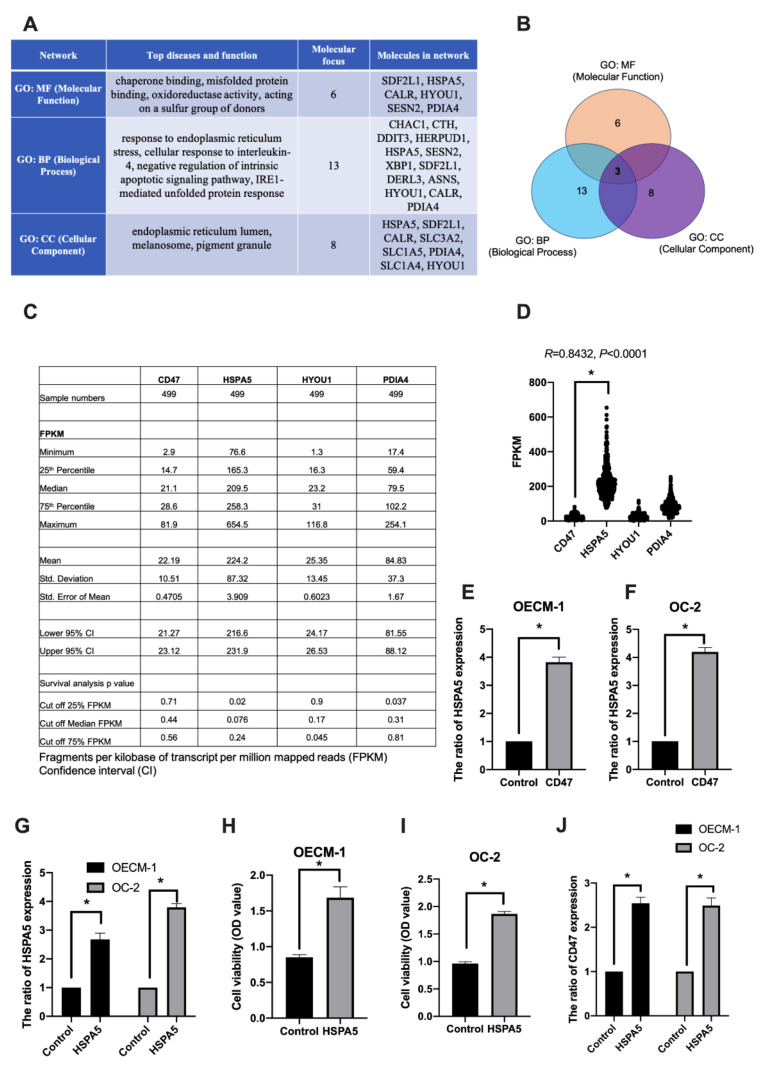
The biofunction pathway analysis of the differentially expressed genes regulated by CD47-overexpression in oral cancer cells. (**A**) The network analysis report of upregulated genes by CD47-overexpression in OECM-1 and OC-2 cells. Molecular focus gene was involved in top diseases and function. (**B**) Three identified genes, HSPA5, HYOU1, and PDIA4, were found to be involved in molecular functions, biological processes, and cellular components in OSCC. (**C**,**D**) CD47, HSPA5, HYOU1, and PDIA4 gene expression and survival was analyzed using the HPA database. Cut off FPKM of 25%, 50%, and 75% were used to find the survival rate, respectively. R; correlation coefficient; two-tailed Student’s *t*-test. (**E**,**F**) The expression of HSPA5 was examined in CD47-overexpressing OECM-1 and OC-2 cells using qPCR. (**G**,**J**) HSPA5 and CD47 gene expression was analyzed by qPCR and (**H**,**I**) cell growth was analyzed by CCK-8 assay. The data are presented as the mean ± SD of three experiments. * *p* < 0.05 vs. empty control; two-tailed Student’s *t*-test.

In addition, we used IPA to analyze the DEGs regulated by CD47-overexpression in oral cancer for network analysis including molecular functions (MFs), biological processes (BPs), and cellular components (CCs). Then, we focused on the upregulated genes by CD47-overexpression in the OECM-1 and OC-2 cell lines. We found six genes (SDF2L1, HSPA5, CALMFR, HYOU1, SESN2, and PDIA4) involved in molecular functions, 13 genes (CHAC1, CTH, DDIT3, HERPUD1, HSPA5, SESN2, XBP1, SDF2L1, DERL3, ASNS, HYOU1, CALR, and PDIA4) involved in biological processes, and eight genes (HSPA5, SDF2L1, CALR, SLC3A2, SLC1A5, PDIA4, SLC1A4, and HYOU1) involved in cellular components (Figure 4A). Furthermore, the results indicate that HSPA5, HYOU1, and PDIA4 genes appear in all three categories (Figure 4B).

To further explore the role of CD47, HSPA5, HYOU1, and PDIA4 genes in cancer, we used the human protein atlas (HPA) database [26] to analyze the expression of these target genes in cancer and their impact on the ability of cells to survive. We know that OSCC occupies a high proportion (90%) of head and neck squamous cell carcinomas [27]. Therefore, we selected head and neck squamous cell carcinoma samples from 499 patients for gene expression and survival analysis using the HPA database. The results showed that the fragments per kilobase per million (FPKM) of CD47, HSPA5, HYOU1, and PDIA4 was 22.19 ± 10.51, 224.2 ± 87.32, 25.35 ± 13.45, 84.83 ± 37.3, respectively. The increased expression of HSPA5 (cut off quantile 25%), HYOU1 (cut off quantile 75%), and PDIA4 (cut off quantile 25%) was significantly associated with survival rate in all 499 patients (Figure 4C). In addition, HSPA5 was highly expressed in cancer and positively and significantly correlated with CD47 (R = 0.8432, *p* < 0.0001, Figure 4D).

To prove that HSPA5 was associated with CD47-overexpression in the OECM-1 and OC-2 cell lines, we used qPCR to quantify gene expression. We found that the expression of HSPA5 was induced by CD47-overexpression in the OECM-1 (Figure 4E) and OC-2 (Figure 4F) cell lines. We also verified the effect of HSPA5 overexpression in OSCC cell lines. HSPA5 plasmid was transfected into the cells and HSPA5 expression was analyzed by qPCR (Figure 4G). In addition, cell viability assay revealed that HSPA5 increased cell growth in the OECM-1 and OC-2 cell lines (Figure 4H,I). Next, we analyzed the CD47 expression in HSPA5-overexpressing OSCC cell lines. The results found that CD47 expression was induced by HSPA5-overexpression in both in cell lines (Figure 4J). These data suggest that HSPA5, HYOU1 and PDIA4 were significantly associated with survival rate and HSPA5 is a suitable prognostic marker with CD47 in cancer survivability.

### 3.4. The DAVID Analyses of Differentially Expressed Genes following CD47-Overexpression

**Figure 5:**
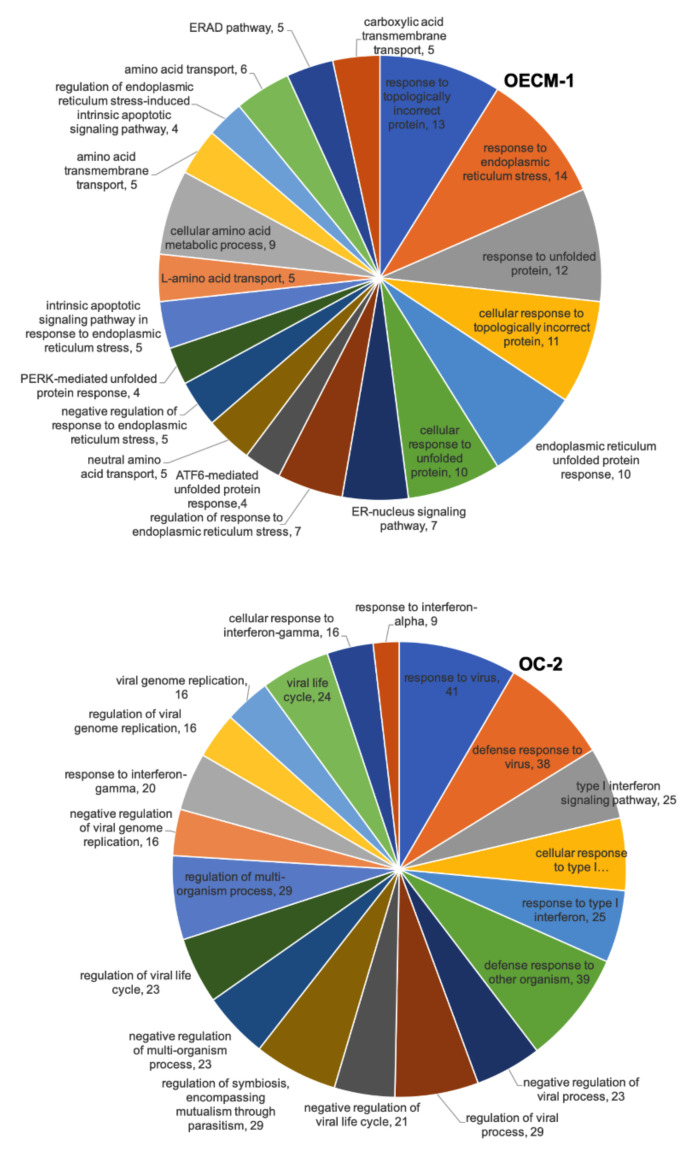
RNA sequencing showing the biofunction of DEGs in CD47-overexpression in the OECM-1 and OC-2 cell lines according to DAVID biological process analysis. The numbers represent the number of genes involved in the biological process.

We also used the DAVID biological process analysis to analyze DEGs with CD47-overexpressing OECM-1 and OC-2 cell lines from RNA sequencing. We concluded that the top 20 biological processes of the DEGs in the OECM-1 and OC-2 cell lines and the top five biological processes of these genes include responses to topologically incorrect proteins (13 genes), endoplasmic reticulum stress (14 genes), and unfolded proteins (12 genes), as well as cellular responses to topologically incorrect proteins (11 genes), endoplasmic reticulum unfolded protein responses (10 genes), and endoplasmic reticulum unfolded protein responses (10 genes) in OECM-1 cells. Meanwhile, in OC-2 cells, these genes include responses to viruses (41 genes), defense responses to other organisms (39 genes), defense responses to the virus (38 genes), the regulation of symbiosis, encompassing mutualism through parasitism (29 genes), and the regulation of viral processes (29 genes) (Figure 5).

## 4. Discussion

Based on previous studies, we know that CD47 is abundantly expressed in many tumors, and it plays an important role in macrophages, affecting the development of cancer [28,29]. In the current study, we identified that the differentially expressed genes in OSCC cells are enriched in the biological functions of CD47, including regulating the response of unfolded proteins by NGS and systematic bioinformatic analysis. The results showed that the unfolded proteins HSPA5, HYOU1, and PDIA4 were associated with survivability in cancer, and HSPA5 was positively and significantly correlated with CD47, and induced by CD47 in OSCC cells lines.

Heat shock protein family A (Hsp70) member 5 (HSPA5) is a member of the molecular chaperones, which regulates the response of unfolded proteins [30]. HSPA5 promotes cancer progression, drug resistance, and metastasis and is a poor prognosis marker [31,32]. Hypoxia and nutrient deprivation in the environment activate the UPR and upregulate HSPA5 expression, leading to the rapid growth of solid tumors [33]. In addition, knockout mouse models have demonstrated that HSPA5 is required for cell proliferation and is critical for embryonic cell growth and survival [34]. In vitro and in vivo studies found that silencing of HSPA5 expression in cancer cells inhibits tumor cell metastasis [35]. Therefore, we believe that HSPA5 plays an important role in cancer progression.

Hypoxia-upregulated protein 1 (HYOU1) belongs to the heat shock protein 70 family, which is overexpressed in many tumors. Highly expressed HYOU1 induces tumor invasion, results in a poor prognosis, and inhibits apoptosis [36,37,38]. A previous study found that heat shock protein modulation primary macrophages are polarized into classic (M1) macrophages and alternative (M2) macrophages. The Hsp70 family might be strictly involved in the effector stages of macrophage activity [39]. Previous research found that anti-CD47 treatment enhanced the phenotype of macrophages converted to M1 subtype, and produced a higher phagocytosis rate through M1 macrophages [40]. In addition, CD47 secreted significant levels of IL-10 via M2 macrophages differentiation to promote cancer cell migration [41]. According to the above results, we believe that CD47 plays an important role in the regulation of HSP. CD47 has the same ability to promote cancer progression [42], drug resistance [43], poor prognosis marker [44] and macrophage polarization [40] as HSP. In addition, CD47 as a transcription factor [45] can mediate gene expression. Our data also found that overexpression of CD47 induced the expression of HSPA5, and overexpression of HSPA5 induced the expression of CD47 in the OECM-1 and OC-2 cells. A previous study also demonstrated that inhibition of glucose-regulated protein 78 (GRP78), a member of the HSP70 family, downregulated CD47 expression in tumor cells [46]. Therefore, we believe that CD47 and HSPA5 have a feedback loop in OSCC cell lines, and suggest that CD47 mediates cancer progression through the HSPA5 signaling pathway in oral squamous cell carcinoma. In the future, we will further explore the mechanisms and regulatory capabilities of CD47 and HSPA5.

PDIA4, as a member of the protein disulfide isomerases (PDI) family, is a key factor for protein folding in the endoplasmic reticulum. A previous study found that PDIA4 participates in tumor progression by influencing cell apoptosis and DNA repair mechanisms [47]. Meanwhile, a recent study pointed out that PDIA4 may be regarded as a biomarker for the treatment of ovarian cancer [48]. Interestingly, PDIA4 has similar properties to HSP proteins, and both have the function of affecting protein folding. Protein folding is important for CD47 evasion of macrophage phagocytosis [49]. However, the detailed mechanism is unclear at present.

## 5. Conclusions

These results provide important insights into possible new diagnostic genes for OSCC-targeting CD47. Although the mechanism by which CD47 regulates unfolded proteins is still unclear, the detailed signaling pathway will be discussed in more depth in the future.

## Figures and Tables

**Table 1:** Genes with significant changes induced by the overexpression of CD47 in OECM-1 and OC-2 cell lines.

Official Gene Symbol	Gene Name	OECM-1-CD47/OECM-1-Empty Control FOLD-Change	OC2-CD47/OC2-Empty Control Fold-Change	GENE Expression
BAALC-AS1	BAALC antisense RNA 1	12.15	6.26	Up
CCDC184	Coiled–coil domain containing 184	5.46	8.88	Up
CRB1	Crumbs 1, cell polarity complex component	5.46	6.26	Up
HPCA	Hippocalcin	7.69	8.88	Up
LINC01099	Long intergenic non-protein coding RNA 1099	7.73	6.26	Up
LINC01512	Long intergenic non-protein coding RNA 1512	5.46	6.26	Up
LOC102723703	Uncharacterized LOC102723703	7.55	8.88	Up
NMBR	Neuromedin B receptor	5.46	6.26	Up
PCP2	Purkinje cell protein 2	5.05	6.26	Up
RASGRP1	RAS guanyl releasing protein 1	5.46	8.88	Up
SMC2-AS1	SMC2 antisense RNA 1 (head to head)	9.92	6.26	Up
SMN2	Survival of motor neuron 2, centromeric	190.25	22.01	Up
TRIM6-TRIM34	TRIM6-TRIM34 readthrough	117.78	48.70	Up
ZC3H12B	Zinc finger CCCH-type containing 12B	5.48	6.26	Up
ZNF32-AS2	ZNF32 antisense RNA 2	7.69	6.26	Up
ZNF664-FAM101A	NA	10.03	8.99	Up
C16orf71	Chromosome 16 open reading frame 71	0.19	0.12	Down
CELF5	CUGBP Elav-like family member 5	0.14	0.16	Down
FAM53B-AS1	FAM53B antisense RNA 1	0.19	0.12	Down
GAL3ST2	Galactose-3-*O*-sulfotransferase 2	0.14	0.16	Down
KATNBL1P6	Katanin regulatory subunit B1-like 1 pseudogene 6	0.11	0.11	Down
LOC100506136	NA	0.14	0.05	Down
LOC101926897	NA	0.20	0.16	Down
LOC101927178	Uncharacterized LOC101927178	0.15	0.12	Down
LOC101927666	Uncharacterized LOC101927666	0.20	0.09	Down
MOG	Myelin oligodendrocyte glycoprotein	0.11	0.16	Down
NRN1L	Neuritin 1 like	0.19	0.12	Down
PROSER2-AS1	PROSER2 antisense RNA 1	0.14	0.12	Down
RALGAPA1P	Ral GTPase activating protein catalytic alpha subunit 1 pseudogene 1	0.01	0.02	Down
RGPD6	RANBP2-like and GRIP domain containing 6	0.12	0.20	Down
RIMS4	Regulating synaptic membrane exocytosis 4	0.19	0.16	Down
THRIL	TNF- and HNRNPL-related immunoregulatory long non-coding RNA	0.19	0.16	Down

Head-to-head: a genomic locus where two adjacent genes are differentially transcribed from opposite DNA strands. Readthrough: continuous transcription. NA: not applicable.

## Data Availability

The data presented in this study are available on request from the corresponding author.
